# South Korea is the center for the origin and emanation of soybean mosaic virus with Bayesian phylogeographic inference

**DOI:** 10.1128/spectrum.02868-24

**Published:** 2025-05-30

**Authors:** Shiqing Wei, Lei Zhang, Liang Cheng, Linwen Liu, Guoliang Chen, Hui Yang, Xiaoyan Qiu, Liya Luo, Guoshu Gong, Min Zhang

**Affiliations:** 1 College of Agronomy, Sichuan Agricultural University 670358 Chengdu, Sichuan, China; 2 CAS Key Laboratory of Pathogen Microbiology and Immunology, Institute of Microbiology, Chinese Academy of Sciences 85387, , Beijing, China; National Taiwan University, Taipei, Taiwan

**Keywords:** soybean mosaic virus, phylogeography, ancestral location, virus dispersal

## Abstract

Soybean mosaic virus (SMV) is one of the most serious viral pathogens, causing reductions in soybean yield worldwide. Using more than 350 time-stamped SMV coat protein encoding gene sequences from more than a dozen countries and regions in Central Asia, East Asia, North America, South America, and Western Europe, we investigated the phylodynamics of SMV to understand the origin and dispersal of the virus via Bayesian phylogeographic inference. Our analysis revealed that the most recent common SMV ancestor occurred in approximately 1511 (95% credibility interval, 1075–1848) Common Era, and the evolutionary rate of the coat protein gene was 3.751 × 10^−4^ substitutions/site/year (95% credibility interval, 2.694 × 10^−4^–4.879 × 10^−4^). Our results suggest that the SMV population may have originated in South Korea and that South Korea has been the major center for the dissemination of SMV. The virus first began to emigrate from Korea to China in the late 17th century, and it took more than 150 years to spread from South Korea to Japan. However, after the 20th century, SMV spread from South Korea to South America, North America, and Western Europe. Furthermore, our results suggest that the migration history of SMV may be related to important human historical events.

**IMPORTANCE:**

Soybean mosaic virus (SMV) is a pathogen that severely affects soybean production areas around the world and can cause up to an 86% reduction in soybean yield. This article provides a comprehensive reconstruction of the phylogeographic history of SMV. Our results revealed the geographic origin and migration history of SMV on a global scale and that the migration history of SMV is correlated with human factors. These results have important implications for the sustainable management of soybean production in the field.

## INTRODUCTION

Soybean (*Glycine max* (L.) Merr.) is one of the most important oil crops worldwide, with an annual production of approximately 370 million tons (United States Department of Agriculture, 2023). Soybean mosaic virus (SMV) was first reported in South Korea in the 1970s (1) and has become a pathogen that severely affects soybean production areas around the world, causing yield reductions of 8%–35% (2). In uniformly infected field plots, SMV can cause up to an 86% loss in soybean yield (3–8). After infection, the leaves of soybean plants are mosaiced, deformed, and necrotic; additionally, the plants are dwarf, and seeds exhibit mottling, even leading to the death of infected soybean plants (9). In the field, SMV is naturally transmitted by noncolonizing aphids in a nonpersistent manner (2). In addition, seeds are one of the transmission modes of SMV in the field, but the efficiency of seed transmission is influenced by the interaction between a specific SMV strain and a soybean line (10, 11).

SMV belongs to the genus *Potyvirus* in the family *Potyviridae* (12). This genus has a single positive-sense RNA genome of ~9.6 kb in length and is encapsidated by the coat protein (CP) to form filamentous virions (9, 13). The genome encodes a large open reading frame, which is translated into a large polyprotein that, upon proteolysis, produces 10 different functional proteins: P1, helper component-proteinase (HC-Pro), P3, 6K1, cylindrical inclusion protein (CI), 6K2, genome-linked viral protein (VPg), nuclear inclusion protein a-proteinase (NIa-Pro), nuclear inclusion protein b (NIb), and CP. The P3 gene coincides with a gene encoding a 25 kDa short polypeptide protein movement protein called PIPO, which is fused to the N-terminus of the P3 protein and is translated by a +2 frame shift (14–16). CI is required for genome replication and movement, and the 6K1 protein helps virions move between cells (17, 18). The P1 protein is a protease and determines the host range of SMV (19). The HC-Pro protein helps virions move long distances and inhibits RNA silencing in the host plant (20, 21). HC-Pro and CP proteins can interact with and participate in aphid transmission by potyviruses (22, 23). In addition, CP acts as a cell-to-cell movement protein (24). VPg binds specifically to eIF4E to initiate polyprotein translation (25), and NIb is a subunit of RNA-dependent RNA polymerase (26, 27).

SMVs can be categorized into seven major strains designated G1–G7 in North America and South Korea (28–31), while in Japan, SMVs can be separated into five strains from A to E (32). In China, SMVs can be categorized into two groups: 22 SC strains (SC1–SC22) identified nationwide and 3 N strains (N1–N3) prevalent in Northeast China (33–36). Phylogenetic analysis revealed that SC7 is closely related to G1 and G3, whereas the other SCs are not related to G1–G7 (37). A recent study also revealed that SC18 is closely related to N strains (38).

Epidemiology is very important for disease prevention and control. With the development of various relevant interdisciplinary endeavors over the past decade, phylodynamics has been used to infer the epidemiological processes of viruses via statistical methods (39–41). Owing to limitations in hosts and transmission vectors, plant viruses cannot spread efficiently across geographical barriers as can animal viruses. However, SMV is globally widespread and causes losses in agricultural production. An increasing number of reports have shown that the dispersal of plant viruses across geographical isolation zones is caused by human factors (42–45). Previous studies have suggested that SMV originated in South Asia and East Asia, particularly in China (38, 46–50). In the present study, the CP gene of SMV was used to study the phylodynamics of the viruses to determine their origin and dispersal. Our Bayesian phylogenetic analysis revealed that SMV originated in South Korea in the 16th century and subsequently spread to other countries in Asia, Europe, North America, and South America.

## MATERIALS AND METHODS

### Recombination analyses

The complete genome sequence of the CP gene of SMV was obtained from GenBank (Table S1). Codon-based sequence multiple alignment was conducted with MAFFT v7 software (51). To detect recombinants in the CP data set, we performed recombination with seven algorithms (RDP, GENECONV, BOOTSCAN, MAXCHI, CHIMAERA, SISCAN, and 3SEQ) in the RDP 4.95 suite (52). Only recombination events detected by at least four of the seven algorithms with an associated *P* value of 10^−5^ were accepted as true recombinants. We removed isolates with no collection date and recombination, ultimately obtained 396 sequences for phylogenetic analysis (Table S1).

### Tests for temporal signals

To assess the clock-like behavior of our data set, we first confirmed the presence of the temporal signal via a clustered date-randomization test recommended by Duchene (53), and we found that our data set has temporal signals (Fig. S1). Prior to this, we used the Mantel test to assess the correlation between pairwise genetic distances and differences in sampling dates (54), and we found no evidence of confounding temporal and genetic structure in our data set (*P* = 0.272; Fig. S1). We then evaluated the temporal structure of all the data sets via the newly developed Bayesian evaluation of temporal signal (BETS; 55), which introduces a heterogeneous model (*M*_het_) and an isochronous model (*M*_iso_) into the same data set, and the marginal likelihood values estimated via generalized stepping stone sampling (56) were used to compare the fits of two competing data sets. The best fit model of the data set was selected via the Bayesian factor (BF) (57). The (log) BF log[*P*(Y|*M*_het_)]−log[*P*(Y|*M*_iso_)] of our data set was greater than 160 (Fig. S1), indicating that there was a sufficient temporal signal in the data. In addition, we calculated the correlation coefficients (*r*) of a regression of the root-to-tip genetic distance against the sampling time with TempEst (58) and used maximum likelihood analysis in IQ-TREE (59). The substitution model for the CP gene is GTR + I + *G*_4_, which was selected via ModelFinder (60) implemented in PhyloSuite (61) based on the Bayesian information criterion. Our results revealed a weak correlation between tip date and genetic distance (*r*^2^ = 9.583 × 10^−3^), suggesting that there were various clock rates among lineages in our data set.

### Temporal dynamics of soybean mosaic virus

The best-fit substitution tree prior and molecular clock models for the data set were calculated in BEAST 1.10 (62) via path sample-based marginal likelihood estimation (63), which consists of Bayesian skyline coalescent models and Gamma relaxed clocks (Table S2). All Markov chain Monte Carlo runs were conducted for 200 million steps, and samples were collected every 20,000 steps. Using Tracer 1.71 (64) to check for convergence, only effective sample sizes above 200 were accepted for all the parameters, after which the first 10% of the samples were removed.

### Discrete phylogeographic analyses

Each geographic region had at least five isolates; therefore, our data set had 11 geographic regions (Canada, Eastern China, North China, Northeast China, Southwest China, Colombia, Iran, Japan, South Korea, and the United States). On the basis of the substitution rate obtained for the CP gene (Table S3), a uniform prior was selected for the calibration of the data set for lineage geographic analysis. Dispersal patterns were inferred with an asymmetric diffusion model and evaluated via Bayesian stochastic search variable selection (65). The rates of viral migration across the discrete locations were calculated from the resulting log file in SpreaD3 (66). Significant migration pathways were determined based on a BF >3 and a mean indicator >0.5. The categories of rate support were as follows: decisively supported diffusion, BF >1,000; very strongly supported diffusion, 1,000 > BF ≥ 150; strongly supported diffusion, 150 > BF ≥ 10; and supported diffusion, 10 > BF ≥ 3 (40). In addition, due to the imbalance in the number of isolates between regions, five randomly sampled subdatasets were created to verify the accuracy and stability of the results obtained from the raw data.

## RESULTS

### The phylogeography of soybean mosaic virus

**Fig 1:**
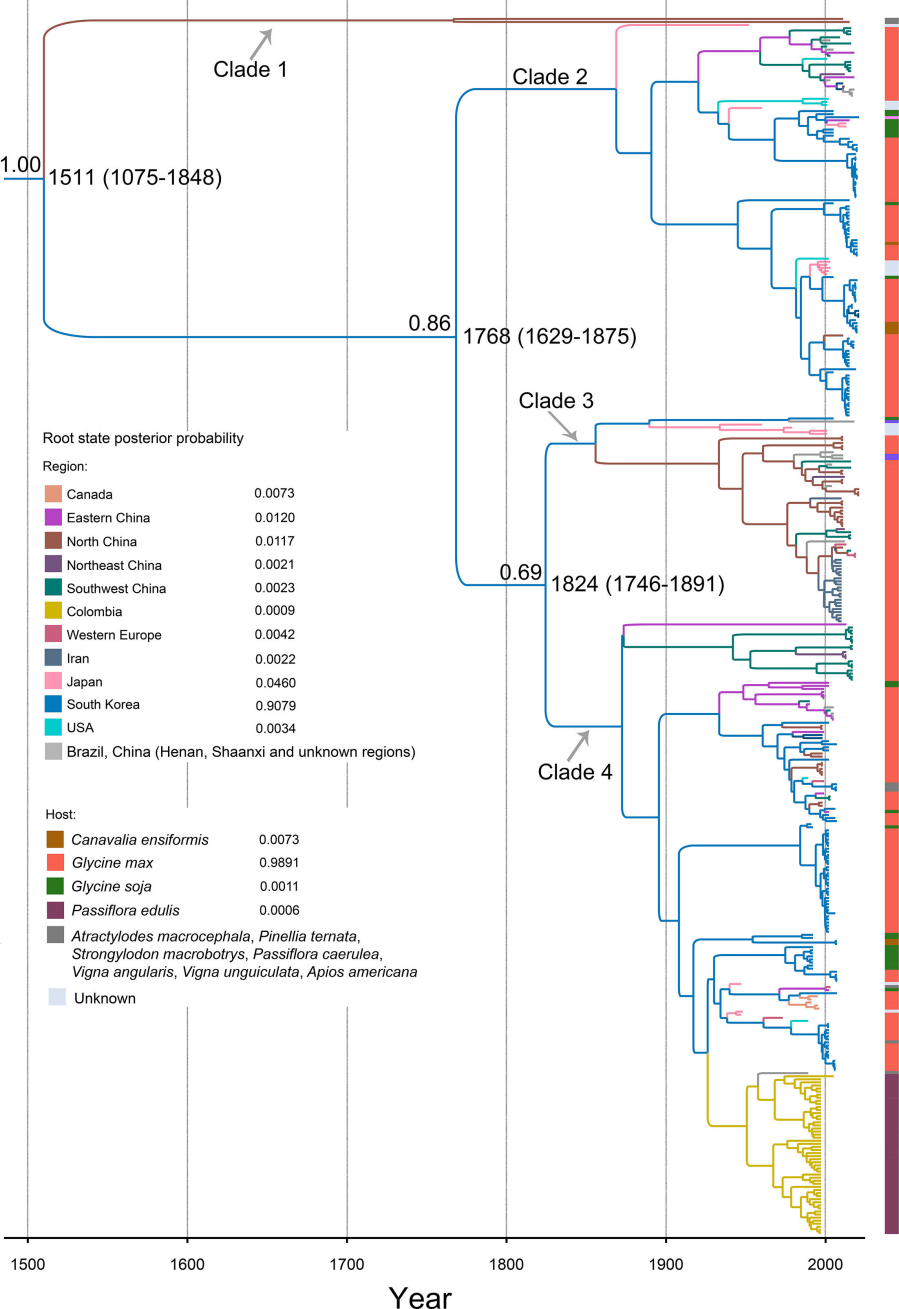
Time-scaled maximum clade credibility tree inferred from the coat protein sequences of soybean mosaic virus. The tree topologies have been chosen to maximize the product of node posterior probabilities. Branch lengths are scaled according to time, as shown by the horizontal axis. Branch colors represent inferred location states. The inset panel shows the root state posterior probabilities of the geographic regions.

Our time-scaled maximum clade credibility (MCC) tree revealed that the evolutionary rate of the CP gene of SMV was 3.751 × 10^−4^ substitutions/site/year (95% credibility interval, 2.694 × 10^−4^–4.879 × 10^−4^), and the virus diverged into four clades at approximately 1511 (95% credibility interval 1075–1848). Clade 1 contained only two isolates amplified from *Pinellia ternata* and *Atractylodes macrocephala* from Shaanxi, China (Fig. 1). The other isolates were divided into three major clades at approximately 1768 (95% credibility interval 1629–1875) and 1824 (95% credibility interval 1746–1891), all of which contained isolates from South Korea (Fig. 1). The isolates of clade 2 are from China, Japan, South Korea, and the United States, whereas the isolates of clade 3 are from China, Japan, Korea, and Iran (Fig. 1). The isolates of clade 4 were obtained from a wide range of regions, including Brazil, Canada, China, Colombia, Japan, South Korea, Western Europe, and the United States (Fig. 1). Notably, the isolates from Iran and Colombia are grouped separately into clade 3 and clade 4 (Fig. 1). With the exception of isolates from Colombia, observing the evolution of SMV related to its geographical distribution and hosts is difficult (Fig. 1).

Our results show that the ancestral state of the SMV was placed in South Korea (root posterior probability = 0.908), which was determined via the Bayesian structured coalescent approximation approach (67). Five subdatasets for the CP gene confirmed these results separately (Table S3). Moreover, the spatial origins of SMV clade 2, clade 3, and clade 4 were all located in South Korea (root posterior probabilities = 0.849, 0.915, and 0.824, respectively; Fig. 1). In addition, we obtained statistics on the host spatial origin of SMV, and the results revealed that the earliest host of SMV was soybean (root posterior probability = 0.989; Fig. 1). The two isolates in clade 1 were from *P. ternata* and *A. macrocephala*, which may affect the host origin of SMV. After these two isolates were removed, we conducted another statistical analysis of the host origin of SMV, and the results revealed that the host origin of SMV was still soybean (root posterior probability = 0.991).

### Dispersal patterns of soybean mosaic virus

**Fig 2:**
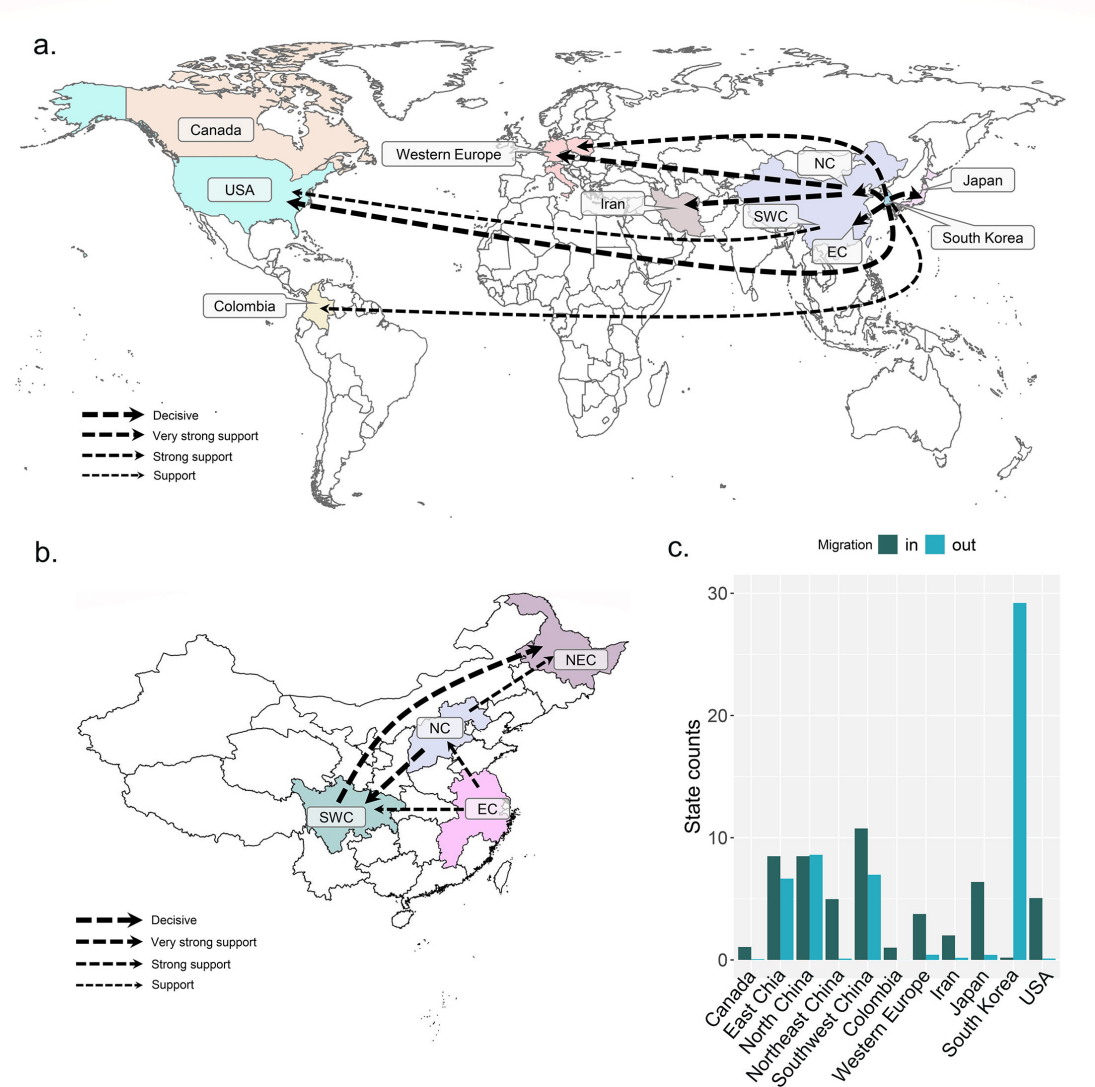
The spatial dynamics analysis of soybean mosaic virus inferred from coat protein. (a) Supported global spatial diffusion pathways and (b) internal pathways within China and (c) histogram of the total number of location-state transitions. EC, East China; NEC, Northeast China; NC, North China; and SWC, Southwest China.

Our Bayesian phylogeographic analysis based on the CP gene of SMV supported 14 migration pathways, including 10 migration pathways from East Asia to Central Asia, North America, South America, and Western Europe (Fig. 2a, Table S3). There are seven migration pathways from South Korea to Colombia, East China, Japan, North China, Western Europe, and the United States (Fig. 2a). The other three migration pathways from East Asia are two migration pathways from North China to Western Europe and Iran, and one migration pathway from East China to the United States (Fig. 2a, Table S3). There were two migration pathways from North China to Southwest China and Northeast China, and two migration pathways from East China to North China and Southwest China. There was a migration pathway from Southwest China to Northeast China (Fig. 2a, Table S4). We reconstructed the spatial spread patterns through five bootstrap replicates using the raw data to determine the accuracy and stability of the results caused by the imbalance in the number of isolates between regions (Table S4). Our results suggested that all the subdatasets stably confirmed the results of the original data sets. However, there is disagreement about the pathway of migration to Canada. The SMV population of Canada from South Korea was supported by raw data, whereas all five subdatasets support a migration pathway from Europe to Canada (Table S4).

**Fig 3:**
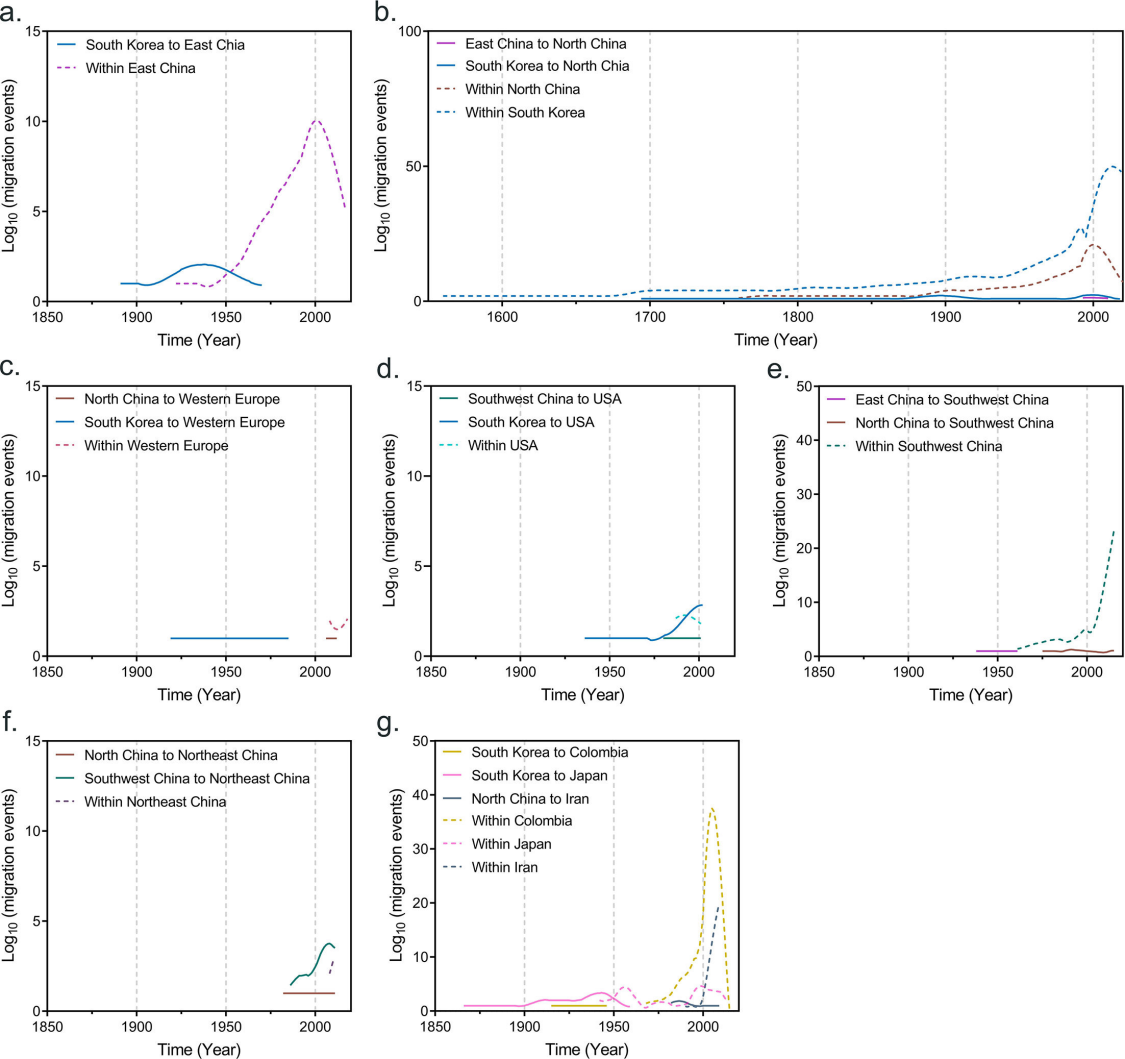
Inferred migration events (on the log_10_ scale) of soybean mosaic virus over time (year) with each region. (a) Migration events immigrate to and within East China. (b) Migration events immigrate to North China and within North China and South Korea. (c) Migration events immigrate to and within Western Europe. (d) Migration events immigrate to and within the United States. (e) Migration events immigrate to and within Southwest China. (f) Migration events immigrate to and within Northeast China. (g) Migration events immigrate to and within Canada, Colombia, Japan, and Iran.

Our results suggest that SMV first spread via cross-regional transmission from South Korea to North China in the late 17th century (Fig. 3b) and that SMV from South Korea again spread to China approximately 200 years later (Fig. 3a). The virus of China first spread via cross-regional transmission from East China to Southwest China in approximately 1938, and the SMV of China subsequently spread via cross-regional transmission from North China to Southwest China in approximately 1975 (Fig. 3e). The SMV population of Southwest China began to expand in approximately 1962 and spread to the United States and Northeast China in approximately 1980 and 1985, respectively (Fig. 3d and f). Otherwise, the population of SMVs from Northeast China first emigrated from North China in approximately 1981, and a second emigration from Southwest China occurred in approximately 1984 (Fig. 3f). Moreover, the SMV population of Iran also emigrated from North China in approximately 1982 (Fig. 3g). The SMV from Western Europe spread from South Korea and North China in approximately 1919 and 2004 (Fig. 3c). The first spread of SMV from Korea to the Americas was to Colombia in approximately 1914, followed by the United States in approximately 1936 (Fig. 3g). In addition, the SMV in Japan came from South Korea in 1865 (Fig. 3g). In all the cases examined, intra-country/zone migrations of SMV were detectable, and high rates of intra-country/zone migrations were observed in Colombia, East China, Iran, North China, Southwest China, and South Korea (Fig. 3a, b, e, and g).

Consistent with the origin location of the SMV, South Korea was found to be a major source for SMV emigrations, and almost no SMVs immigrated to South Korea (Fig. 2c). Colombia, Iran, Japan, Northeast China, and the United States experienced almost no SMV emigration (Fig. 2c). In addition, North China, East China, and Southwest China are important SMV emigration regions, and immigration to these places is relatively notable (Fig. 2c).

## DISCUSSION

By screening all the complete CP gene sequences retrieved from GenBank, we conducted a large-scale systematic dynamic analysis of the global SMV population. Previous studies have suggested that SMV may have originated in South Asia and East Asia (46–50), and a recent study implies that SMV possibly originated in China (38). However, our results suggest that soybean mosaic virus originated in South Korea in the early 16th century. This finding is not surprising, as SMV was first discovered and reported in South Korea in the 1970s (1). Our results indicate that South Korea is the most important region for SMV emigration in the world, which is surprising given that South Korea is also a major importer of soybean, despite it being the second largest crop after rice in terms of acreage (68). Moreover, North America and South America are the major exporter regions of soybean (Food and Agriculture Organization of the United Nations, FAO), but our phylogeographic analysis results revealed that SMV in both the United States and Colombia came from South Korea. Moreover, our MCC tree suggested that SMV in Brazil may also have spread from South Korea (Fig. 1).

The SMV of China from South Korea first spread in the late 17th century. In the late 17th century, North China, especially Beijing, was the political and economic center of the Qing Dynasty. At this time, the Korean Peninsula and the Qing Dynasty were very allied, and frequent trade exchanges may have allowed SMV to spread to North China by accident. However, the population of SMVs in China began to expand in the middle of the 20th century, which may be related to the founding of the People’s Republic of China. From the beginning of the Opium War in 1840 to the founding of the People’s Republic of China in 1949, the war on this land ended, which meant stable and gradual development of agricultural production and economic activity. Stable and increasing soybean agricultural production has provided conditions for the expansion of the SMV population. This may be one of the reasons why the migration of SMV within China occurred mainly after approximately 1980, as China implemented a policy of reform and opening up in 1978. The first emigration of SMV within China was from East China to Southwest China in approximately 1938, while in 1937, the Republic of China government moved from Nanjing in East China to Chongqing in Southwest China after being defeated by Japan (modern history.org.cn). The relocation of the capital was accompanied by many personnel and material transfers, including political, economic, and educational transfers, which may have led to the relocation of the SMV from East China to Southwest China. There have been reports that SMV from Iran emigrated from East Asia (69, 70), and our results further confirm and clarify that the virus emigrated from North China in approximately 1982. There was an emigration pathway from Southwest China to the United States in approximately 1980. This is not surprising, as it was only in 1979 and 1971 that China formally established diplomatic relations with the United States and Iran (Ministry of Foreign Affairs of the People’s Republic of China). In addition, the spread of SMV from North China to Western Europe occurred in 2004, which may be related to China joining the World Trade Organization in 2001 (Ministry of Commerce of the People’s Republic of China).

Recombination is an important factor in the evolution and diversity of RNA viruses (71). Soybean mosaic viruses exhibit very common intraspecific and interspecific recombination (70–75). A special interspecific recombinant strain formed from bean common mosaic virus and SMV is widely distributed in southern China (73, 75). SMV Pinellia isolates are caused by the recombination of SMV and dasheen mosaic virus in the P1 gene (76). These reports suggest that recombination is an important factor in the evolution of SMV. The classification of SMV strains in different regions differs based on the resistance of different soybean varieties to SMV. In North America and South Korea, SMV can be categorized into seven major pathotypes (31), and in China, SMV has been identified in 22 strains (33–36), whereas in Japan, SMV has been identified in 5 strains (32). Phylogenetic relationships reconstructed from the P1 gene of SMV have indicated that the evolution of SMV may be geographically related, whereas the phylogenetic tree reconstructed from the CP gene was independent of geography (69). Although the isolates from Iran and Colombia were clustered in clade 3 and clade 4, respectively, and the isolates from China, Japan, South Korea, Western Europe, and the United States were scattered across the clades, indicating that there were no evolutionary or geographical associations. In addition, our results revealed that the clustering of CP genes was independent of the host. The P1 protein is involved in the adaptation of SMV to the host (19). Moreover, strains of SMVs in different regions are divided based on the response of different soybean varieties to the virus (28–36). Therefore, in addition to recombination, we believe that the evolution and diversity of SMV are related to host and geographically driven adaptations.

In summary, we have obtained fresh insights into the worldwide origin and dispersal of SMV via Bayesian phylogeographic inference. Our phylogeographic analysis indicates that the SMV population may have originated in South Korea, and South Korea has been the major center of SMV transmission for 300 years, with China being another important center of spread for SMV. Our results show that SMV from South Korea began to spread to China in the late 17th century and has been spreading in East Asia for approximately the subsequent 200 years. After the 20th century, SMV from East Asia gradually spread to South America, North America, and Central Asia. Furthermore, our results show that the global migration pathways and history of the SMV may be related to major human historical events.

## Data Availability

We guarantee the authenticity and availability of all data and materials in the manuscript, and the results/data/figures in this manuscript have not been published elsewhere, nor are they under consideration (from you or one of your contributing authors) by another publisher.

## SUPPLEMENTAL MATERIAL

Fig. S1 spectrum.02868-24-s0001.tif Results of tests for the temporal signal in the data set.

Supplemental material spectrum.02868-24-s0002.docx Fig. S1 caption.

Supplemental tables spectrum.02868-24-s0003.docx Tables S1 to S4.

ASM does not own the copyrights to Supplemental Material that may be linked to, or accessed through, an article. The authors have granted ASM a non-exclusive, world-wide license to publish the Supplemental Material files. Please contact the corresponding author directly for reuse.
