# Supplementary material for: South Korea is the center for the origin and emanation of soybean mosaic virus with Bayesian phylogeographic inference
Source: Microbiol Spectr. 2025 May 30;13(7):e02868-24. doi: 10.1128/spectrum.02868-24 (PMC12211032; doi:10.1128/spectrum.02868-24)

Supplementary Figure caption:

Results of tests for the temporal signal in the dataset. (a) Mantel test of confounding of genetic and temporal distances. The *y*-axis indicates differences in sampling years and the *x*-axis shows genetic distance. (b) Date-randomization test for temporal signal in the sequence data. The *y*-axis shows the substitution rate and the *x*-axis shows 10 different randomizations of the dates after clustered permutations in the dataset.

Supplementary Figure:


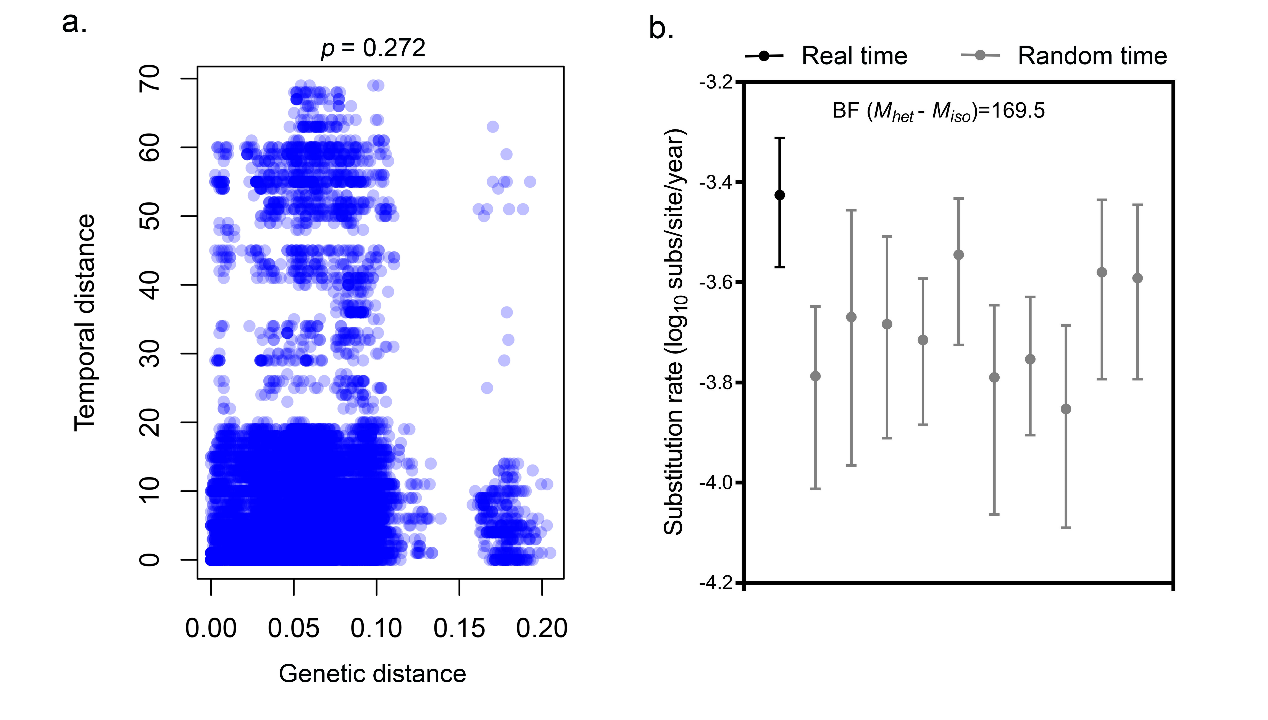

Supplement: Supplemental material — Fig. S1 caption. [file spectrum.02868-24-s0002.docx]
