# Supplementary material for: South Korea is the center for the origin and emanation of soybean mosaic virus with Bayesian phylogeographic inference
Source: Microbiol Spectr. 2025 May 30;13(7):e02868-24. doi: 10.1128/spectrum.02868-24 (PMC12211032; doi:10.1128/spectrum.02868-24)
Supplement: Supplemental tables — Tables S1 to S4. [file spectrum.02868-24-s0003.docx]

Supplementary Material

Supplementary Fig. 1 Results of tests for the temporal signal in the dataset. (a) Mantel test of confounding of genetic and temporal distances. The y-axis indicates differences in sampling years and the x-axis shows genetic distance. (b) Date-randomization test for temporal signal in the sequence data. The *y*-axis shows the substitution rate and the *x*-axis shows 10 different randomizations of the dates after clustered permutations in the dataset.

Supplementary Table. 1 Isolates of soybean mosaic virus used in this study.

| Accession number | Location | Collecting year | Original host |
| --- | --- | --- | --- |
| MN124783 | Brazil | 2003 | *Passiflora sp.* |
| MW300425 | Brazil | Mar-2019 | *Strongylodon macrobotrys* |
| EU871724 | Canada | Aug-2005 | *Glycine max* |
| EU871725 | Canada | Aug-2005 | *Glycine max* |
| JN416770 | Canada | 01-Sep-2010 | *Glycine max* |
| China | | | |
| KR065437 | Hebei Province | 10-Aug-2012 | *Glycine max* |
| KR065473 | Hebei Province | 07-Aug-2012 | *Glycine max* |
| KR065474 | Hebei Province | 07-Aug-2012 | *Glycine max* |
| KR065475 | Hebei Province | 07-Aug-2012 | *Glycine max* |
| KR065476 | Hebei Province | 07-Aug-2012 | *Glycine max* |
| KR065477 | Hebei Province | 07-Aug-2012 | *Glycine max* |
| KR065479 | Hebei Province | 07-Aug-2012 | *Glycine max* |
| KR065480 | Hebei Province | 07-Aug-2012 | *Glycine max* |
| KR065481 | Hebei Province | 07-Aug-2012 | *Glycine max* |
| KR065482 | Hebei Province | 10-Aug-2012 | *Glycine max* |
| KR065483 | Hebei Province | 07-Aug-2012 | *Glycine max* |
| KR065484 | Hebei Province | 07-Aug-2012 | *Glycine max* |
| KR065485 | Hebei Province | 07-Aug-2012 | *Glycine max* |
| KR065486 | Hebei Province | 07-Aug-2012 | *Glycine max* |
| KR065487 | Hebei Province | 07-Aug-2012 | *Glycine max* |
| KR065488 | Hebei Province | 07-Aug-2012 | *Glycine max* |
| KR065489 | Hebei Province | 07-Aug-2012 | *Glycine max* |
| KR065490 | Hebei Province | 07-Aug-2012 | *Glycine max* |
| KR065491 | Hebei Province | 07-Aug-2012 | *Glycine max* |
| KR065492 | Hebei Province | 07-Aug-2012 | *Glycine max* |
| KR065493 | Hebei Province | 07-Aug-2012 | *Glycine max* |
| KR065494 | Hebei Province | 07-Aug-2012 | *Glycine max* |
| KR065495 | Hebei Province | 07-Aug-2012 | *Glycine max* |
| KR065496 | Hebei Province | 07-Aug-2012 | *Glycine max* |
| KR065497 | Hebei Province | 07-Aug-2012 | *Glycine max* |
| KR065498 | Hebei Province | 07-Aug-2012 | *Glycine max* |
| KR065499 | Hebei Province | 07-Aug-2012 | *Glycine max* |
| KR065500 | Hebei Province | 07-Aug-2012 | *Glycine max* |
| KR065501 | Hebei Province | 07-Aug-2012 | *Glycine max* |
| KR065502 | Hebei Province | 07-Aug-2012 | *Glycine max* |
| KR065503 | Hebei Province | 07-Aug-2012 | *Glycine max* |
| KR065504 | Hebei Province | 07-Aug-2012 | *Glycine max* |
| KR065505 | Hebei Province | 07-Aug-2012 | *Glycine max* |
| KR065506 | Hebei Province | 07-Aug-2012 | *Glycine max* |
| OR514681 | Hebei Province | Aug-2022 | *Glycine max* |
| OR514682 | Hebei Province | Aug-2022 | *Glycine max* |
| KR065507 | Hebei Province | 07-Aug-2012 | *Glycine max* |
| MW364642 | Hebei Province | 20-Nov-2020 | *Pinellia ternata* |
| OR514683 | Hebei Province | Aug-2022 | *Glycine max* |
| OR514684 | Hebei Province | Aug-2022 | *Glycine max* |
| OR514685 | Hebei Province | Aug-2022 | *Glycine max* |
| OR514686 | Hebei Province | Aug-2022 | *Glycine max* |
| MW354949 | Shandong Province | 18-Sep-2019 | *Glycine max* |
| KP710869 | Northeast | Jun-2013 | *Glycine max* |
| KP710871 | Heilongjiang Province | Jun-2013 | *Glycine max* |
| KP710872 | Heilongjiang Province | Jun-2013 | *Glycine max* |
| KP710873 | Heilongjiang Province | Jun-2013 | *Glycine max* |
| KP710874 | Heilongjiang Province | Jun-2013 | *Glycine max* |
| KP710875 | Heilongjiang Province | Jun-2013 | *Glycine max* |
| KP710876 | Heilongjiang Province | Jun-2013 | *Glycine max* |
| KP710877 | Heilongjiang Province | Jun-2013 | *Glycine max* |
| KP710878 | Heilongjiang Province | Jun-2013 | *Glycine max* |
| KC818636 | Shaanxi Province | 01-May-2012 | *Vigna unguiculata (Linn.) Walp* |
| KC845322 | Shaanxi Province | 01-Jul-2012 | *Atractylodes macrocephala* |
| KC896416 | Shaanxi Province | 01-May-2012 | *Vigna unguiculata (Linn.) Walp* |
| KP710870 | Shaanxi Province | Jun-2013 | *Glycine max* |
| MT712111 | Shanxi Province | 18-Aug-2016 | *Pinellia ternata* |
| KP710861 | Jiangxi Province | Jun-2013 | *Glycine max* |
| MW354948 | Jiangsu Province | 18-Sep-2019 | *Glycine max* |
| OK105105 | Jiangsu Province | 06-May-2019 | *Glycine max* |
| KR024718 | Jiangsu Province | 03-Sep-2014 | *Glycine max* |
| KT285170 | Jiangsu Province | 10-Jun-2004 | *Glycine max* |
| KX834319 | Jiangsu Province | 04-Jan-2016 | *Glycine soja* |
| KX834320 | Jiangsu Province | 04-Jan-2016 | *Glycine soja* |
| KX834322 | Jiangsu Province | 04-Jan-2016 | *Glycine soja* |
| KX834323 | Jiangsu Province | 04-Jan-2016 | *Glycine soja* |
| KX834321 | Jiangsu Province | 04-Jan-2016 | *Glycine soja* |
| KX834324 | Jiangsu Province | 04-Jan-2016 | *Glycine soja* |
| KX834325 | Jiangsu Province | 04-Jan-2016 | *Glycine soja* |
| KP710862 | Jiangxi Province | Jun-2013 | *Glycine max* |
| KP710863 | Jiangxi Province | Jun-2013 | *Glycine max* |
| KP710864 | Jiangxi Province | Jun-2013 | *Glycine max* |
| KP710865 | Jiangxi Province | Jun-2013 | *Glycine max* |
| KP710866 | Jiangxi Province | Jun-2013 | *Glycine max* |
| KP710867 | Sichuan Province | Jun-2010 | *Glycine max* |
| HM590055 | Chongqing | May-2004 | *Glycine max* |
| HM590054 | Sichuan Province | Mar-2004 | *Glycine max* |
| MH389868 | Sichuan Province | Sep-2017 | *Glycine max* |
| MH389869 | Sichuan Province | Sep-2017 | *Glycine max* |
| MH389870 | Sichuan Province | Sep-2017 | *Glycine max* |
| MH389871 | Sichuan Province | Sep-2017 | *Glycine max* |
| MH389872 | Sichuan Province | Sep-2017 | *Glycine max* |
| MH389873 | Sichuan Province | Sep-2017 | *Glycine max* |
| MH389874 | Sichuan Province | Sep-2017 | *Glycine max* |
| MH389875 | Sichuan Province | Sep-2017 | *Glycine max* |
| MH389876 | Sichuan Province | Sep-2017 | *Glycine max* |
| MH389877 | Sichuan Province | Sep-2017 | *Glycine max* |
| MH389878 | Sichuan Province | Sep-2017 | *Glycine max* |
| MH389879 | Sichuan Province | Sep-2017 | *Glycine max* |
| MH389880 | Sichuan Province | Sep-2017 | *Glycine max* |
| MH389881 | Sichuan Province | Sep-2017 | *Glycine max* |
| MH389882 | Sichuan Province | Sep-2017 | *Glycine max* |
| MH389883 | Sichuan Province | Sep-2017 | *Glycine max* |
| MH389884 | Sichuan Province | Sep-2017 | *Glycine max* |
| MH389885 | Sichuan Province | Sep-2017 | *Glycine max* |
| MH389886 | Sichuan Province | Sep-2017 | *Glycine max* |
| MH389887 | Sichuan Province | Sep-2017 | *Glycine max* |
| MH389888 | Sichuan Province | Sep-2017 | *Glycine max* |
| MH389889 | Sichuan Province | Sep-2017 | *Glycine max* |
| MH389890 | Sichuan Province | Sep-2017 | *Glycine max* |
| MH389891 | Sichuan Province | Sep-2017 | *Glycine max* |
| MH389892 | Sichuan Province | Sep-2017 | *Glycine max* |
| MH389893 | Sichuan Province | Sep-2017 | *Glycine max* |
| MH389894 | Sichuan Province | Sep-2017 | *Glycine max* |
| MH389895 | Sichuan Province | Sep-2017 | *Glycine max* |
| MH389896 | Sichuan Province | Sep-2017 | *Glycine max* |
| MH919384 | Sichuan Province | 2017 | *Glycine max* |
| MH919385 | Sichuan Province | 2017 | *Glycine max* |
| MH919386 | Sichuan Province | 2017 | *Glycine max* |
| MN539670 | Sichuan Province | 2002 | *Glycine max* |
| MW354946 | Anhui Province | 18-Sep-2019 | *Glycine max* |
| AJ628750 | Zhejiang Province | Unknown | *Pinellia ternata* |
| OK625818 | Zhejiang Province | 09-Dec-2020 | *Glycine max* |
| MW354950 | Henan Province | 18-Sep-2019 | *Glycine max* |
| MN623289 | Unknown | 2018 | *Glycine max* |
| MN623290 | Unknown | 2018 | *Glycine max* |
| HQ396721 | Unknown | 2006 | *Glycine max* |
| HQ396722 | Unknown | 2004 | *Glycine max* |
| HQ396723 | Unknown | 2004 | *Glycine max* |
| HQ396724 | Unknown | 2006 | *Glycine max* |
| HQ396725 | Unknown | 2004 | *Glycine max* |
| JF833013 | Unknown | 2005 | *Glycine max* |
| JF833014 | Guangdong Province | 2005 | *Glycine max* |
| JF833015 | Guangxi Province | 2005 | *Glycine max* |
| MK350280 | Liaoning Province | 15-Jul-2017 | *Glycine max* |
| MW354947 | Unknown | 18-Sep-2019 | *Glycine max* |
| MW354951 | Unknown | 18-Sep-2019 | *Glycine max* |
| LC037232 | Taiwan | 30-Jun-2014 | *Chenopodium quinoa* |
| KX096578 | Unknown | 28-Jan-2015 | *Glycine max* |
| OK058515 | Unknown | Unknown | *Nicotiana benthamiana* |
| OR607764 | Unknown | Unknown | *Nicotiana benthamiana* |
| AJ310200 | Unknown | Unknown | *Glycine max* |
| AJ312439 | Zhejiang Province | Unknown | *Glycine max* |
| JQ361514 | Colombia | 01-Nov-2011 | *Passiflora edulis* |
| JQ361515 | Colombia | 01-Nov-2011 | *Passiflora edulis* |
| JQ361516 | Colombia | 01-Nov-2011 | *Passiflora edulis* |
| JQ361517 | Colombia | 01-Nov-2011 | *Passiflora edulis* |
| JQ361518 | Colombia | 01-Nov-2011 | *Passiflora edulis* |
| JQ361519 | Colombia | 01-Nov-2011 | *Passiflora edulis* |
| JQ361520 | Colombia | 01-Nov-2011 | *Passiflora edulis* |
| JQ361521 | Colombia | 01-Nov-2011 | *Passiflora edulis* |
| JQ361522 | Colombia | 01-Nov-2011 | *Passiflora edulis* |
| JQ361523 | Colombia | 01-Nov-2011 | *Passiflora edulis* |
| JQ361524 | Colombia | 01-Nov-2011 | *Passiflora edulis* |
| JQ361525 | Colombia | 01-Nov-2011 | *Passiflora edulis* |
| JQ361526 | Colombia | 01-Nov-2011 | *Passiflora edulis* |
| JQ361527 | Colombia | 01-Nov-2011 | *Passiflora edulis* |
| JQ361528 | Colombia | 01-Nov-2011 | *Passiflora edulis* |
| JQ361529 | Colombia | 01-Nov-2011 | *Passiflora edulis* |
| JQ361530 | Colombia | 01-Nov-2011 | *Passiflora edulis* |
| JQ361531 | Colombia | 01-Nov-2011 | *Passiflora edulis* |
| JQ361532 | Colombia | 01-Nov-2011 | *Passiflora edulis* |
| JQ361533 | Colombia | 01-Nov-2011 | *Passiflora edulis* |
| JQ361534 | Colombia | 01-Nov-2011 | *Passiflora edulis* |
| JQ361535 | Colombia | 01-Nov-2011 | *Passiflora edulis* |
| JQ361536 | Colombia | 01-Nov-2011 | *Passiflora edulis* |
| JQ361537 | Colombia | 01-Nov-2011 | *Passiflora edulis* |
| JQ361538 | Colombia | 01-Nov-2011 | *Passiflora edulis* |
| JQ361539 | Colombia | 01-Nov-2011 | *Passiflora edulis* |
| JQ361540 | Colombia | 01-Nov-2011 | *Passiflora edulis* |
| JQ361541 | Colombia | 01-Nov-2011 | *Passiflora edulis* |
| JQ361542 | Colombia | 01-Nov-2011 | *Passiflora edulis* |
| JQ361543 | Colombia | 01-Nov-2011 | *Passiflora edulis* |
| JQ361544 | Colombia | 01-Nov-2011 | *Passiflora edulis* |
| JQ361545 | Colombia | 01-Nov-2011 | *Passiflora edulis* |
| JQ361546 | Colombia | 01-Nov-2011 | *Passiflora edulis* |
| JQ361547 | Colombia | 01-Nov-2011 | *Passiflora edulis* |
| JQ361548 | Colombia | 01-Nov-2011 | *Passiflora edulis* |
| JQ361549 | Colombia | 01-Nov-2011 | *Passiflora edulis* |
| JQ361550 | Colombia | 01-Nov-2011 | *Passiflora edulis* |
| JQ361551 | Colombia | 01-Nov-2011 | *Passiflora edulis* |
| JQ361552 | Colombia | 01-Nov-2011 | *Passiflora edulis* |
| JQ361553 | Colombia | 01-Nov-2011 | *Passiflora edulis* |
| JQ361554 | Colombia | 01-Nov-2011 | *Passiflora edulis* |
| JQ361555 | Colombia | 01-Nov-2011 | *Passiflora edulis* |
| JQ361556 | Colombia | 01-Nov-2011 | *Passiflora edulis* |
| JQ361557 | Colombia | 01-Nov-2011 | *Passiflora edulis* |
| JQ361558 | Colombia | 01-Nov-2011 | *Passiflora edulis* |
| JQ361559 | Colombia | 01-Nov-2011 | *Passiflora edulis* |
| JQ361560 | Colombia | 01-Nov-2011 | *Passiflora edulis* |
| JQ361561 | Colombia | 01-Nov-2011 | *Passiflora edulis* |
| JQ361562 | Colombia | 01-Nov-2011 | *Passiflora edulis* |
| JQ361563 | Colombia | 01-Nov-2011 | *Passiflora edulis* |
| JQ361564 | Colombia | 01-Nov-2011 | *Passiflora edulis* |
| KY249378 | Colombia | 01-Aug-2015 | *Passiflora edulis f. edulis* |
| MW655827 | Colombia | 2019 | *Passiflora edulis f. edulis* |
| OL875089 | Colombia | 2021 | *Passiflora edulis* |
| OP564897 | Colombia | Unknown | *Yellow Passion fruit* |
| MN399738 | Germany | 2017 | *legume* |
| ON013906 | Germany | Unknown | *Glycine max* |
| OR526468 | Italy | 07-Sep-1987 | *Glycine max* |
| MW822167 | Netherlands | Jul-2020 | *Glycine max* |
| MW822168 | Netherlands | Jul-2020 | *Glycine max* |
| KM886929 | Poland | Jul-2014 | *Glycine max* |
| KM886930 | Poland | Nov-2013 | *Glycine max* |
| KM979229 | India | 29-May-2014 | *Glycine max* |
| KF135467 | Iran | Aug-2011 | *Glycine max* |
| KF135468 | Iran | Aug-2011 | *Glycine max* |
| KF135469 | Iran | Aug-2011 | *Glycine max* |
| KF135470 | Iran | Aug-2011 | *Glycine max* |
| KF135471 | Iran | Aug-2011 | *Glycine max* |
| KF135472 | Iran | Aug-2011 | *Glycine max* |
| KF135473 | Iran | Aug-2011 | *Glycine max* |
| KF135474 | Iran | Aug-2011 | *Glycine max* |
| KF135475 | Iran | Aug-2011 | *Glycine max* |
| KF135476 | Iran | Aug-2011 | *Glycine max* |
| KF135477 | Iran | Aug-2011 | *Glycine max* |
| KF135478 | Iran | Aug-2011 | *Glycine max* |
| KF135479 | Iran | Aug-2011 | *Glycine max* |
| KF135480 | Iran | Aug-2011 | *Glycine max* |
| KF135481 | Iran | Aug-2011 | *Glycine max* |
| KF135482 | Iran | Aug-2011 | *Glycine max* |
| KF135483 | Iran | Aug-2011 | *Glycine max* |
| KF135484 | Iran | Aug-2011 | *Glycine max* |
| KF135485 | Iran | Aug-2011 | *Glycine max* |
| KF135486 | Iran | Aug-2011 | *Glycine max* |
| KF135487 | Iran | Aug-2011 | *Glycine max* |
| KF135488 | Iran | Aug-2011 | *Glycine max* |
| MW464638 | Iran | 01-Oct-2020 | *Glycine max* |
| LC268997 | Japan | 2014-11-04 | *Glycine soja* |
| LC268998 | Japan | 2014-10-07 | *Glycine soja* |
| LC323107 | Japan | 1961 | *Glycine max* |
| AB100444 | Japan | 1953 | *Unknown* |
| AB100445 | Japan | 1961 | *Unknown* |
| AB100446 | Japan | 1961 | *Unknown* |
| AB100447 | Japan | 1961 | *Unknown* |
| AB100448 | Japan | 1962 | *Unknown* |
| AB206827 | Japan | 2002 | *Unknown* |
| AB206828 | Japan | 2002 | *Unknown* |
| AB206829 | Japan | 2003 | *Unknown* |
| AB206830 | Japan | 2002 | *Unknown* |
| AB206831 | Japan | 2002 | *Unknown* |
| AB206832 | Japan | 2003 | *Unknown* |
| AB206833 | Japan | 2004 | *Unknown* |
| AB206834 | Japan | 2004 | *Unknown* |
| AB100442 | Japan | Unknown | *Unknown* |
| AB100443 | Japan | Unknown | *Unknown* |
| FJ548849 | South Korea | Jul-2006 | *wild soybean* |
| FJ640954 | South Korea | Jun-2006 | *wild soybean* |
| FJ640955 | South Korea | Jun-2006 | *wild soybean* |
| FJ640956 | South Korea | Jun-2006 | *wild soybean* |
| FJ640957 | South Korea | Jun-2006 | *wild soybean* |
| FJ640958 | South Korea | Jun-2006 | *wild soybean* |
| FJ640959 | South Korea | Jun-2006 | *wild soybean* |
| FJ640960 | South Korea | Jun-2006 | *wild soybean* |
| FJ640961 | South Korea | Jun-2006 | *wild soybean* |
| FJ640962 | South Korea | Jun-2006 | *wild soybean* |
| FJ640963 | South Korea | Jun-2006 | *wild soybean* |
| FJ640964 | South Korea | Jun-2006 | *wild soybean* |
| FJ640965 | South Korea | Jun-2006 | *wild soybean* |
| FJ640966 | South Korea | Jul-2006 | *wild soybean* |
| FJ640967 | South Korea | Jul-2006 | *wild soybean* |
| FJ640968 | South Korea | Jul-2006 | *wild soybean* |
| FJ640969 | South Korea | Jul-2006 | *wild soybean* |
| FJ640970 | South Korea | Jul-2006 | *wild soybean* |
| FJ640971 | South Korea | Jul-2006 | *wild soybean* |
| FJ640972 | South Korea | Jul-2006 | *wild soybean* |
| FJ640973 | South Korea | Jul-2006 | *wild soybean* |
| FJ640974 | South Korea | Jul-2006 | *wild soybean* |
| FJ640975 | South Korea | Jul-2006 | *wild soybean* |
| FJ640976 | South Korea | Jul-2006 | *wild soybean* |
| FJ640981 | South Korea | Oct-2003 | *Glycine max* |
| FJ807700 | South Korea | Jul-2005 | *Glycine max* |
| FJ807701 | South Korea | 2005 | *Glycine max* |
| AY294044 | South Korea | 2001 | *Glycine max* |
| AY294045 | South Korea | Unknown | *Glycine max* |
| KY986929 | South Korea | 25-Jul-2016 | *Vigna angularis* |
| LC591946 | South Korea | 2019-10-17 | *Glycine max* |
| LC655955 | South Korea | 2019-08-28 | *Glycine max* |
| LC655957 | South Korea | 2021-08-11 | *Glycine max* |
| LC655958 | South Korea | 2021-08-11 | *Glycine max* |
| LC735724 | South Korea | 2022-08-04 | *Phaseolus vulgaris* |
| LC761388 | South Korea | 2022-10-28 | *Glycine max* |
| LC786829 | South Korea | 2020-09-16 | *Glycine max* |
| MK561002 | South Korea | 04-Sep-2018 | *Glycine max* |
| MK561003 | South Korea | 04-Sep-2018 | *Glycine max* |
| MK561004 | South Korea | 04-Sep-2018 | *Glycine max* |
| MT603826 | South Korea | Jun-2016 | *Glycine max* |
| MT603828 | South Korea | Jun-2016 | *Glycine max* |
| MT603829 | South Korea | Jun-2016 | *Glycine max* |
| MT603830 | South Korea | Jun-2016 | *Glycine max* |
| MT603831 | South Korea | Jun-2016 | *Glycine max* |
| MT603832 | South Korea | Jun-2016 | *Glycine max* |
| MT603833 | South Korea | Jun-2016 | *Glycine max* |
| MT603834 | South Korea | Jun-2016 | *Glycine max* |
| MT603835 | South Korea | Jun-2016 | *Glycine max* |
| MW079179 | South Korea | Jul-2020 | *Glycine max* |
| MW079180 | South Korea | Jul-2020 | *Glycine max* |
| MW079181 | South Korea | Jul-2020 | *Glycine max* |
| MW079182 | South Korea | Jul-2020 | *Glycine max* |
| MW079183 | South Korea | Jul-2020 | *Glycine max* |
| MW079184 | South Korea | Jul-2020 | *Glycine max* |
| MW079185 | South Korea | Jul-2020 | *Glycine max* |
| MW079186 | South Korea | Jul-2020 | *Glycine max* |
| MW079187 | South Korea | Jul-2020 | *Glycine max* |
| MW079188 | South Korea | Jul-2020 | *Glycine max* |
| MW079189 | South Korea | Jul-2020 | *Glycine max* |
| MW079190 | South Korea | Jul-2020 | *Glycine max* |
| MW079191 | South Korea | Jul-2020 | *Glycine max* |
| MW079192 | South Korea | Jul-2020 | *Glycine max* |
| MW079193 | South Korea | Jul-2020 | *Glycine max* |
| MW079194 | South Korea | Jul-2020 | *Glycine max* |
| MW079195 | South Korea | Jul-2020 | *Glycine max* |
| MW079196 | South Korea | Jul-2020 | *Glycine max* |
| MW079197 | South Korea | Jul-2020 | *Glycine max* |
| MW079198 | South Korea | Jul-2020 | *Glycine max* |
| MW079199 | South Korea | Jul-2020 | *Glycine max* |
| MW079200 | South Korea | Jul-2020 | *Glycine max* |
| MW079201 | South Korea | Jul-2020 | *Glycine max* |
| OK155984 | South Korea | 12-Aug-2021 | *Glycine max* |
| OK155985 | South Korea | 28-Jul-2021 | *Glycine max* |
| OK155986 | South Korea | 28-Jul-2021 | *Glycine max* |
| OK155987 | South Korea | 12-Aug-2021 | *Glycine max* |
| OK155988 | South Korea | 12-Aug-2021 | *Glycine max* |
| OK155989 | South Korea | 12-Aug-2021 | *Glycine max* |
| ON676526 | South Korea | Jul-2019 | *Glycine max* |
| ON843744 | South Korea | 01-Jun-2021 | *Glycine max* |
| ON843745 | South Korea | 01-Jun-2021 | *Glycine max* |
| ON843746 | South Korea | 01-Jun-2021 | *Glycine max* |
| ON843747 | South Korea | 01-Jun-2021 | *Glycine max* |
| ON843748 | South Korea | 01-Jun-2021 | *Glycine max* |
| ON843749 | South Korea | 01-Jun-2021 | *Glycine max* |
| ON843750 | South Korea | 01-Jun-2021 | *Glycine max* |
| OP046403 | South Korea | 10-Jul-2021 | *Canavalia ensiformis* |
| OP046404 | South Korea | 10-Jul-2021 | *Canavalia ensiformis* |
| OP046405 | South Korea | 10-Jul-2021 | *Canavalia ensiformis* |
| OP046406 | South Korea | 10-Jul-2021 | *Canavalia ensiformis* |
| OP046407 | South Korea | 10-Jul-2021 | *Canavalia ensiformis* |
| OP046408 | South Korea | 10-Jul-2021 | *Canavalia ensiformis* |
| OP046409 | South Korea | 10-Jul-2021 | *Canavalia ensiformis* |
| OP046410 | South Korea | 10-Aug-2021 | *Apios americana* |
| OP046411 | South Korea | 10-Aug-2021 | *Apios americana* |
| OP046412 | South Korea | 10-Aug-2021 | *Apios americana* |
| OP389994 | South Korea | Jun-2016 | *Glycine max* |
| OP389995 | South Korea | Jun-2016 | *Glycine max* |
| OP389996 | South Korea | Jun-2016 | *Glycine max* |
| OP389997 | South Korea | Jun-2016 | *Glycine max* |
| OP389998 | South Korea | Jun-2016 | *Glycine max* |
| OP389999 | South Korea | Jun-2016 | *Glycine max* |
| OP390000 | South Korea | Jun-2016 | *Glycine max* |
| OP390001 | South Korea | Jun-2016 | *Glycine max* |
| OP390002 | South Korea | Jun-2016 | *Glycine max* |
| OP390003 | South Korea | Jun-2016 | *Glycine max* |
| OP390004 | South Korea | Jun-2016 | *Glycine max* |
| OP390005 | South Korea | Jun-2016 | *Glycine max* |
| OP390006 | South Korea | Jun-2016 | *Glycine max* |
| OP390007 | South Korea | Jun-2016 | *Glycine max* |
| OP390008 | South Korea | Jun-2016 | *Glycine max* |
| OP390009 | South Korea | Jun-2016 | *Glycine max* |
| OP390010 | South Korea | Jun-2016 | *Glycine max* |
| OP390011 | South Korea | Jun-2016 | *Glycine max* |
| OP390012 | South Korea | Jun-2016 | *Glycine max* |
| OP390013 | South Korea | Jun-2016 | *Glycine max* |
| OP390014 | South Korea | Jun-2016 | *Glycine max* |
| OP390015 | South Korea | Jun-2016 | *Glycine max* |
| OP390016 | South Korea | Jun-2016 | *Glycine max* |
| OP390017 | South Korea | Jun-2016 | *Glycine max* |
| OP390018 | South Korea | Jun-2016 | *Glycine max* |
| OP390019 | South Korea | Jun-2016 | *Glycine max* |
| OP390020 | South Korea | Jun-2016 | *Glycine max* |
| OP390021 | South Korea | Jun-2016 | *Glycine max* |
| OP390022 | South Korea | Jun-2016 | *Glycine max* |
| OP390023 | South Korea | Jun-2016 | *Glycine max* |
| OP390024 | South Korea | Jun-2016 | *Glycine max* |
| OP390025 | South Korea | Jun-2016 | *Glycine max* |
| OP390026 | South Korea | Jun-2016 | *Glycine max* |
| OP390028 | South Korea | Jun-2016 | *Glycine max* |
| OP390030 | South Korea | Jun-2016 | *Glycine max* |
| OP390031 | South Korea | Jun-2016 | *Glycine max* |
| OP390032 | South Korea | Jun-2016 | *Glycine max* |
| OP390033 | South Korea | Jun-2016 | *Glycine max* |
| OP390034 | South Korea | Jun-2016 | *Glycine max* |
| OP390035 | South Korea | Jun-2016 | *Glycine max* |
| OP390036 | South Korea | Jun-2016 | *Glycine max* |
| OP390037 | South Korea | Jun-2016 | *Glycine max* |
| OP390038 | South Korea | Jun-2016 | *Glycine max* |
| OP390039 | South Korea | Jun-2016 | *Glycine max* |
| OP390040 | South Korea | Jun-2016 | *Glycine max* |
| OP390041 | South Korea | Jun-2016 | *Glycine max* |
| OP390042 | South Korea | Jun-2016 | *Glycine max* |
| OP390043 | South Korea | Jun-2016 | *Glycine max* |
| OP390044 | South Korea | Jun-2016 | *Glycine max* |
| OP390045 | South Korea | Jun-2016 | *Glycine max* |
| OP390046 | South Korea | Jun-2016 | *Glycine max* |
| OP390047 | South Korea | Jun-2016 | *Glycine max* |
| OP390048 | South Korea | Jun-2016 | *Glycine max* |
| OP390049 | South Korea | Jun-2016 | *Glycine max* |
| OP390050 | South Korea | Jun-2016 | *Glycine max* |
| OP390051 | South Korea | Jun-2016 | *Glycine max* |
| OP390052 | South Korea | Jun-2016 | *Glycine max* |
| OP390053 | South Korea | Jun-2016 | *Glycine max* |
| OP390055 | South Korea | Jun-2016 | *Glycine max* |
| OP390056 | South Korea | Jun-2016 | *Glycine max* |
| OP390058 | South Korea | Jun-2016 | *Glycine max* |
| OP390059 | South Korea | Jun-2016 | *Glycine max* |
| OP390060 | South Korea | Jun-2016 | *Glycine max* |
| OP390062 | South Korea | Jun-2016 | *Glycine max* |
| OP390063 | South Korea | Jun-2016 | *Glycine max* |
| OP390064 | South Korea | Jun-2016 | *Glycine max* |
| OP390065 | South Korea | Jun-2016 | *Glycine max* |
| OP390066 | South Korea | Jun-2016 | *Glycine max* |
| OP390067 | South Korea | Jun-2016 | *Glycine max* |
| OP390068 | South Korea | Jun-2016 | *Glycine max* |
| OP390069 | South Korea | Jun-2016 | *Glycine max* |
| OP390070 | South Korea | Jun-2016 | *Glycine max* |
| OP390071 | South Korea | Jun-2016 | *Glycine max* |
| OP390072 | South Korea | Jun-2016 | *Glycine max* |
| OP390073 | South Korea | Jun-2016 | *Glycine max* |
| OP390074 | South Korea | Jun-2016 | *Glycine max* |
| OP390075 | South Korea | Jun-2016 | *Glycine max* |
| OP390076 | South Korea | Jun-2016 | *Glycine max* |
| OP961954 | South Korea | Jul-2020 | *Glycine max* |
| OQ161628 | South Korea | Jun-2021 | *Glycine max* |
| OQ161629 | South Korea | Jun-2021 | *Glycine max* |
| OQ161630 | South Korea | Jun-2021 | *Glycine max* |
| OQ161631 | South Korea | Jun-2021 | *Glycine max* |
| OQ161632 | South Korea | Jun-2021 | *Glycine max* |
| OQ161633 | South Korea | Jun-2021 | *Glycine max* |
| OQ161634 | South Korea | Jun-2021 | *Glycine max* |
| OQ161635 | South Korea | Jun-2020 | *Glycine max* |
| OQ161636 | South Korea | Jun-2020 | *Glycine max* |
| OQ161637 | South Korea | Jun-2020 | *Glycine max* |
| OQ161638 | South Korea | Jun-2020 | *Glycine max* |
| OQ161639 | South Korea | Jun-2020 | *Glycine max* |
| AJ619757 | South Korea | Unknown | *Unknown* |
| AY216010 | USA | 2003 | *Glycine max* |
| AY216987 | USA | 2003 | *Glycine max* |
| GU015011 | USA | 2002 | *Glycine max* |
| AF241739 | USA | Unknown | *Unknown* |
| FJ640977 | USA | Oct-2003 | *Glycine max* |
| FJ640978 | USA | Oct-2003 | *Glycine max* |
| FJ640979 | USA | Oct-2003 | *Glycine max* |
| FJ640980 | USA | Oct-2003 | *Glycine max* |
| FJ640982 | USA | Oct-2003 | *Glycine max* |

Supplementary Table. 2 Marginal likelihoods of different combinations of clock models and tree priors.

| Model of rate variation | Coalescent tree prior | Relaxed distribution model | Log marginal likelihood | |
| --- | --- | --- | --- | --- |
|  |  |  | Path sampling | Stepping-stone sampling |
| Strict clock | Bayesian skyline |  | -11666.363 | -11711.715 |
| Strict clock | Constant size |  | -11740.848 | -11789.092 |
| Strict clock | Exponential growth |  | -11739.015 | -11766.974 |
| **Uncorrelated lognormal** **relaxed clock** | **Bayesian skyline** | Lognormal | -11606.467 | -11652.740 |
|  |  | **Gamma** | **-11574.983** | **-11628.907** |
|  |  | Exponential | -11583.417 | -11636.601 |
| Uncorrelated lognormal relaxed clock | Constant size | Lognormal | -11655.899 | -11705.413 |
|  |  | Gamma | -11629.317 | -11675.200 |
|  |  | Exponential | -11632.851 | -11676.846 |
| Uncorrelated lognormal relaxed clock | Exponential growth | Lognormal | -11632.422 | -11683.984 |
|  |  | Gamma | -11643.578 | -11701.078 |
|  |  | Exponential | -11672.664 | -11687.780 |

The best-fitting tree prior and molecular clock model are indicated in bold font.

Supplementary Table 3. Root state posterior probability of different sub-datasets of soybean mosaic virus.

| Root state posterior probability | Dataset | | | | | | |
| --- | --- | --- | --- | --- | --- | --- | --- |
|  | Original data | Bootstrap 1 | Bootstrap 2 | | Bootstrap 3 | Bootstrap 4 | Bootstrap 5 |
| Canada | 0.0073 | 0.0060 | | 0.0122 | 0.0022 | 0.0126 | 0.0014 |
| Eastern China | 0.0120 | 0.0121 | | 0.0437 | 0.0253 | 0.0364 | 0.1487 |
| North China | 0.0117 | 0.0063 | | 0.0069 | 0.0102 | 0.0059 | 0.0086 |
| Northeast China | 0.0021 | 0.0014 | | 0.0026 | 0.0025 | 0.0020 | 0.0024 |
| Southwest China | 0.0023 | 0.0027 | | 0.0024 | 0.0019 | 0.0012 | 0.0040 |
| Colombia | 0.0009 | 0.0006 | | 0.0011 | 0.0006 | 0.0008 | 0.0004 |
| Western Europe | 0.0042 | 0.0021 | | 0.0068 | 0.0050 | 0.0045 | 0.0044 |
| Iran | 0.0022 | 0.0006 | | 0.0014 | 0.0015 | 0.0016 | 0.0011 |
| Japan | 0.0460 | 0.1078 | | 0.0797 | 0.1701 | 0.0423 | 0.1153 |
| South Korea | 0.9079 | 0.8593 | | 0.8405 | 0.7778 | 0.8908 | 0.6752 |
| The Unites States | 0.0034 | 0.0012 | | 0.0027 | 0.0028 | 0.0019 | 0.0025 |

Supplementary Table. 4 Statistically supported migration rates of soybean mosaic virus.

| Dataset | From | To | Indicator *^a^* | Bayes factor *^b^* |
| --- | --- | --- | --- | --- |
| Original data | East China | North China | 31.155 | 0.770 |
|  | East China | Southwest China | 26.258 | 0.739 |
|  | North China | Northeast China | 12.899 | 0.581 |
|  | North China | Southwest China | >1000 | 1.000 |
|  | North China | Western Europe | 17.830 | 0.658 |
|  | North China | Iran | >1000 | 0.996 |
|  | Southwest China | Northeast China | >1000 | 0.998 |
|  | Southwest China | The Unites States | 10.825 | 0.538 |
|  | Western Europe | Canada | 3.278 | 0.267 |
|  | South Korea | Canada | 9.205 | 0.511 |
|  | South Korea | Colombia | 73.505 | 0.888 |
|  | South Korea | East China | >1000 | 0.999 |
|  | South Korea | Japan | >1000 | 1.000 |
|  | South Korea | North China | >1000 | 1.000 |
|  | South Korea | Western Europe | 561.658 | 0.984 |
|  | South Korea | The Unites States | >1000 | 0.999 |
| Bootstrap 1 | East China | North China | 48.176 | 0.838 |
|  | East China | Southwest China | 245.607 | 0.964 |
|  | North China | Northeast China | 10.398 | 0.526 |
|  | North China | Southwest China | 326.132 | 0.972 |
|  | North China | Western Europe | 12.831 | 0.580 |
|  | North China | Iran | >1000 | 0.991 |
|  | Southwest China | Northeast China | 601.339 | 0.985 |
|  | Southwest China | The Unites States | 9.393 | 0.511 |
|  | Western Europe | Canada | 9.815 | 0.522 |
|  | South Korea | Canada | 3.661 | 0.283 |
|  | South Korea | Colombia | 9.795 | 0.520 |
|  | South Korea | East China | 701.482 | 0.987 |
|  | South Korea | Japan | 173.700 | 0.949 |
|  | South Korea | North China | 372.038 | 0.976 |
|  | South Korea | Western Europe | 33.457 | 0.78 |
|  | South Korea | The Unites States | 906.652 | 0.990 |
| Bootstrap 2 | East China | North China | 72.468 | 0.886 |
|  | East China | Southwest China | 803.810 | 0.989 |
|  | North China | Northeast China | 9.616 | 0.517 |
|  | North China | Southwest China | 326.132 | 0.972 |
|  | North China | Western Europe | 10.369 | 0.528 |
|  | North China | Iran | 190.680 | 0.954 |
|  | Southwest China | Northeast China | >1000 | 0.994 |
|  | Southwest China | The Unites States | 10.331 | 0.523 |
|  | Western Europe | Canada | 6.424 | 0.409 |
|  | South Korea | Canada | 3.547 | 0.276 |
|  | South Korea | Colombia | 9.352 | 0.515 |
|  | South Korea | East China | 701.482 | 0.987 |
|  | South Korea | Japan | 241.874 | 0.963 |
|  | South Korea | North China | 372.038 | 0.976 |
|  | South Korea | Western Europe | 29.728 | 0.762 |
|  | South Korea | The Unites States | 431.569 | 0.979 |
| Bootstrap 3 | East China | North China | 72.500 | 0.886 |
|  | East China | Southwest China | 682.653 | 0.987 |
|  | North China | Northeast China | 9.769 | 0.518 |
|  | North China | Southwest China | 418.922 | 0.978 |
|  | North China | Western Europe | 9.352 | 0.519 |
|  | North China | Iran | 241.874 | 0.963 |
|  | Southwest China | Northeast China | >1000 | 0.993 |
|  | Southwest China | The Unites States | 9.969 | 0.524 |
|  | Western Europe | Canada | 9.842 | 0.519 |
|  | South Korea | Canada | 2.046 | 0.182 |
|  | South Korea | Colombia | 10.253 | 0.529 |
|  | South Korea | East China | 217.357 | 0.959 |
|  | South Korea | Japan | 33.889 | 0.785 |
|  | South Korea | North China | 532.076 | 0.983 |
|  | South Korea | Western Europe | 35.098 | 0.791 |
|  | South Korea | The Unites States | 456.116 | 0.980 |
| Bootstrap 4 | East China | North China | 81.472 | 0.898 |
|  | East China | Southwest China | >1000 | 0.995 |
|  | North China | Northeast China | 9.760 | 0.517 |
|  | North China | Southwest China | 795.070 | 0.988 |
|  | North China | Western Europe | 9.512 | 0.513 |
|  | North China | Iran | 378.200 | 0.976 |
|  | Southwest China | Northeast China | 11.616 | 0.548 |
|  | Southwest China | The Unites States | 11.616 | 0.548 |
|  | Western Europe | Canada | 9.338 | 0.509 |
|  | South Korea | Canada | 3.547 | 0.276 |
|  | South Korea | Colombia | 10.004 | 0.521 |
|  | South Korea | East China | 791.275 | 0.988 |
|  | South Korea | Japan | >1000 | 0.992 |
|  | South Korea | North China | >1000 | 0.992 |
|  | South Korea | Western Europe | 34.042 | 0.786 |
|  | South Korea | The Unites States | 716.009 | 0.987 |
| Bootstrap 5 | East China | North China | 98.873 | 0.914 |
|  | East China | Southwest China | >1000 | 0.991 |
|  | North China | Northeast China | 10.814 | 0.530 |
|  | North China | Southwest China | 563.222 | 0.984 |
|  | North China | Western Europe | 9.327 | 0.509 |
|  | North China | Iran | 317.222 | 0.972 |
|  | Southwest China | Northeast China | >1000 | 0.994 |
|  | Southwest China | The Unites States | 9.444 | 0.511 |
|  | Western Europe | Canada | 10.329 | 0.527 |
|  | South Korea | Canada | 1.535 | 0.142 |
|  | South Korea | Colombia | 9.033 | 0.503 |
|  | South Korea | East China | 29.161 | 0.758 |
|  | South Korea | Japan | 57.051 | 0.860 |
|  | South Korea | North China | 112.134 | 0.924 |
|  | South Korea | Western Europe | 27.068 | 0.745 |
|  | South Korea | The Unites States | 143.021 | 0.939 |

*^a^* Decisive rates: BF>1,000; Very strongly supported rates: 100≤BF<1,000; Strongly supported rates: 10≤BF<100; Supported rates: 5≤BF<10.

*^b^* Statistically supported migration rates with a mean indicator of >0.5:
